# Association between cardiometabolic index and mortality risk in patients with depression: A comprehensive analysis based on two national cohorts

**DOI:** 10.1097/MD.0000000000048900

**Published:** 2026-05-22

**Authors:** Yixuan Xie, Jiawang Jin, Ting Zhou

**Affiliations:** a Department of Public Health and Health Management, Gannan Medical University, Ganzhou, China.

**Keywords:** cardiometabolic index, cohort study, cross-cultural study, depression, mortality, prognosis

## Abstract

Depression is associated with increased cardiometabolic morbidity and mortality. However, the prognostic value of integrated biomarkers for mortality risk in this population is underexplored, particularly across different ethnicities. The cardiometabolic index (CMI), a composite measure of central obesity and dyslipidemia, has demonstrated predictive utility in general populations, but its role in depression is unknown. To investigate and validate the association between CMI and mortality risk in adults with depression using 2 large, nationally representative cohorts from the United States and China. This study utilized data from the National Health and Nutrition Examination Survey and the China Health and Retirement Longitudinal Study. CMI was calculated as the waist-to-height ratio multiplied by the triglyceride/high-density lipoprotein–cholesterol ratio. Cox proportional hazards models will be employed to examine the association between CMI and all-cause and cardiovascular mortality in participants with depression, after adjusting for potential confounders. This bi-national study evaluates CMI as a prognostic tool for mortality in the depression population. If its utility is confirmed, CMI could serve as a simple, cost-effective biomarker to identify high-risk individuals, facilitating targeted preventive strategies and improving clinical outcomes.

## 1. Introduction

Depression represents one of the most prevalent mental health disorders globally, affecting approximately 264 million people worldwide and constituting a leading cause of disability across all age groups. The bidirectional relationship between depression and cardiometabolic disorders has been extensively documented in epidemiological studies, with depression patients exhibiting significantly higher rates of diabetes, cardiovascular disease, metabolic syndrome, and associated mortality compared to the general population. This complex interplay between mental health and metabolic dysfunction has prompted researchers to investigate novel integrated biomarkers that can simultaneously capture both metabolic and cardiovascular risk profiles in vulnerable populations.

The cardiometabolic index (CMI), first introduced by Wakabayashi and Daimon, represents an innovative composite measure that integrates central obesity and dyslipidemia components through the mathematical relationship of waist-to-height ratio multiplied by the triglyceride-to-high-density lipoprotein (HDL) cholesterol ratio. Unlike traditional individual risk factors, CMI provides a unified assessment of cardiometabolic burden that has demonstrated superior predictive capability for diabetes mellitus, cardiovascular events, and metabolic syndrome compared to individual components alone. The theoretical foundation of CMI lies in its ability to capture the synergistic effects of abdominal adiposity and atherogenic dyslipidemia, 2 key pathophysiological mechanisms underlying cardiometabolic disease development.

Depression patients represent a particularly vulnerable population for cardiometabolic complications due to multiple converging pathways including chronic inflammation, hypothalamic-pituitary-adrenal axis dysregulation, autonomic nervous system dysfunction, behavioral factors such as physical inactivity and poor dietary habits, and medication-induced metabolic side effects. The prevalence of metabolic syndrome in depression patients ranges from 25% to 47%, substantially higher than in the general population, while cardiovascular mortality rates are elevated by 50% to 100% compared to non-depressed individuals. Despite this well-established association, the prognostic utility of integrated cardiometabolic measures such as CMI in predicting mortality outcomes specifically in depression populations remains underexplored.

Previous studies examining cardiometabolic risk in depression have predominantly focused on individual risk factors or traditional composite measures, with limited investigation of novel integrated indices. Furthermore, most existing research has been constrained by single-population studies, limiting the generalizability of findings across different ethnic and geographic populations. The current investigation addresses these knowledge gaps by conducting a comprehensive analysis utilizing 2 large-scale, nationally representative databases: the National Health and Nutrition Examination Survey (NHANES) from the United States and the China Health and Retirement Longitudinal Study (CHARLS) from China, providing unprecedented scope for cross-cultural validation of CMI’s prognostic utility in depression populations.

## 2. Methods

### 2.1. Study design and data sources

This investigation employed a retrospective cohort design utilizing data from 2 major epidemiological databases. The NHANES database, maintained by the National Center for Health Statistics, represents a continuous, nationally representative survey of the non-institutionalized United States population, employing a complex, multistage probability sampling design to ensure population representativeness. For this analysis, we utilized NHANES cycles from 2005 to 2018, encompassing 14 years of continuous data collection with standardized protocols for demographic, laboratory, anthropometric, and health examination data. The NHANES protocol includes comprehensive medical history interviews, physical examinations conducted in mobile examination centers, and extensive laboratory testing performed under rigorous quality control standards.

The CHARLS database represents a nationally representative longitudinal study of Chinese residents aged 45 years and older, designed to provide comprehensive data on health, retirement, and aging patterns in China. Initiated in 2011, CHARLS employs a multistage stratified probability sampling method covering 150 counties and 450 villages or urban communities across 28 provinces, representing approximately 95% of China’s total population. The study design incorporates standardized questionnaires, physical performance measures, and biomarker collection protocols harmonized with international aging studies including the Health and Retirement Study and the English Longitudinal Study of Ageing (ELSA).

### 2.2. Participant Selection and Inclusion Criteria

The participant selection process for both databases followed rigorous inclusion and exclusion criteria to ensure study population homogeneity and data quality. For the NHANES cohort, the initial sampling frame included 70,190 participants across the 2005 to 2018 cycles. Exclusion criteria were systematically applied, removing 30,441 participants aged <20 years, 36,728 participants with missing depression data or non-depression status, and 1748 participants with missing CMI data, BMI data, or mortality information. The final NHANES analytical sample comprised 1269 participants with confirmed depression diagnosis and complete covariate data.

**Figure 1.**
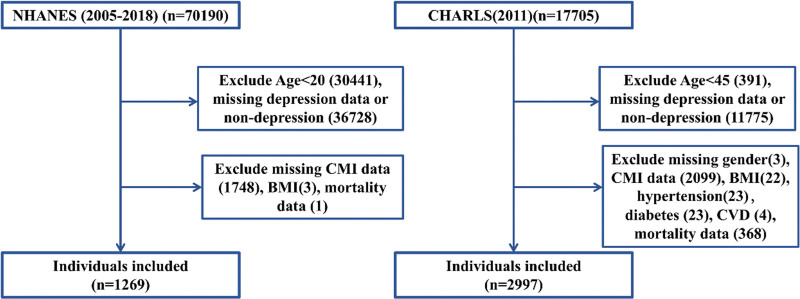
Study flow diagram

For the CHARLS cohort, the initial 2011 baseline sample included 17,705 participants. Systematic exclusions were applied, removing 391 participants aged <45 years, 11,775 participants with missing depression data or non-depression status, and 2542 participants with missing gender, CMI data, BMI, hypertension, diabetes, cardiovascular disease, or mortality data. The final CHARLS analytical sample comprised 2997 participants with confirmed depression diagnosis and complete follow-up data. The complete participant selection flowchart is presented in Figure 1, illustrating the systematic approach to achieving analytically robust study populations.

### 2.3. Cardiometabolic index calculation and depression assessment

The cardiometabolic index was calculated using the validated formula: CMI = waist-to-height ratio (WHtR) × [triglyceride (TG) (mg/dL)/ high-density lipoprotein cholesterol (HDL-C) (mg/dL)], where WHtR represents the waist-to-height ratio calculated as waist circumference (cm) divided by height (cm). Anthropometric measurements in both databases followed standardized protocols with trained technicians using calibrated equipment. Waist circumference was measured at the midpoint between the lowest rib and the iliac crest, while height was measured using stadiometers with participants in stocking feet. Triglyceride and HDL cholesterol levels were measured using standardized enzymatic methods in certified laboratories, with samples collected after appropriate fasting periods according to each database’s protocols.

Depression assessment in NHANES utilized the Patient Health Questionnaire-9 (PHQ-9), a validated self-administered instrument that evaluates 9 depressive symptoms corresponding to Diagnostic and Statistical Manual of Mental Disorders, Fourth Edition (DSM-IV) criteria for major depressive disorder. Each item is scored on a 4-point Likert scale ranging from 0 (“not at all”) to 3 (“nearly every day”), generating total scores from 0 to 27. The PHQ-9 has demonstrated excellent psychometric properties with sensitivity and specificity exceeding 80% for major depression diagnosis when using a cutoff score of 10 or greater. Participants achieving PHQ-9 scores ≥ 10 were classified as having depression, consistent with established clinical practice guidelines.

Depression assessment in CHARLS employed the 10-item Center for Epidemiologic Studies Depression Scale (CES-D-10), a widely validated screening instrument for depressive symptomatology in epidemiological studies. The CES-D-10 evaluates depressive symptoms experienced during the previous week, with response options ranging from “rarely or none of the time” to “most or all of the time.” Items 5 and 8 are reverse-coded, and responses are scored from 0 to 3, generating total scores from 0 to 30. The CES-D-10 has demonstrated robust psychometric properties across diverse populations, with established cutoff scores for identifying clinically significant depressive symptoms. Participants with CES-D-10 scores exceeding 10 were classified as having depression, consistent with validated thresholds used in previous large-scale epidemiological studies.

### 2.4. Mortality ascertainment and follow-up procedures

Mortality outcomes in the NHANES cohort were ascertained through linkage with the National Death Index (NDI), maintained by the National Center for Health Statistics. The NDI represents the most comprehensive source of mortality data in the United States, capturing death certificates from all 50 states and the District of Columbia. Follow-up extended from the date of NHANES examination through December 31, 2019, providing substantial observation periods for mortality assessment. Cause-specific mortality classification utilized International Classification of Diseases, Tenth Revision (ICD-10) codes, with cardiovascular mortality defined as deaths attributed to heart disease (I20-I25, I30-I52) and cerebrovascular disease (I60-I69).

Mortality ascertainment in CHARLS employed a multi-wave follow-up design with mortality information collected through household interviews, village informants, and official death registries. Follow-up assessments were conducted in 2013, 2015, 2018, and 2020, with trained interviewers conducting structured interviews with household members or knowledgeable informants. For deaths occurring between survey waves, detailed information was collected regarding date of death, location, and circumstances. The CHARLS mortality ascertainment protocol has been validated against official death registries, demonstrating high sensitivity and specificity for mortality detection in the Chinese population.

### 2.5. Covariate assessment and variable definition

Comprehensive covariate assessment was conducted to enable appropriate adjustment for potential confounding factors. Demographic variables included age (continuous), gender (male/female), and educational attainment categorized as below high school versus high school or above, reflecting meaningful educational thresholds in both populations. Anthropometric measures included body mass index calculated as weight (kg) divided by height (m) squared, with obesity defined using the standard threshold of ≥ 30 kg/m^2^ for international comparability.

Behavioral risk factors encompassed smoking status and alcohol consumption, both assessed through standardized questionnaires. Smoking status was dichotomized as current/former smoker versus never smoker, while alcohol consumption was classified as current drinker versus non-drinker based on self-reported consumption patterns. These variables were selected based on their established associations with both depression and cardiometabolic outcomes.

Clinical comorbidities included hypertension, diabetes mellitus, and cardiovascular disease, defined using standardized criteria across both databases. Hypertension was defined as self-reported physician diagnosis, measured blood pressure ≥ 140/90 mm Hg, or current use of antihypertensive medications. Diabetes mellitus definition incorporated self-reported physician diagnosis, elevated fasting glucose (≥126 mg/dL), elevated hemoglobin A1c (≥6.5%), or current use of antidiabetic medications. Cardiovascular disease encompassed self-reported physician diagnosis of coronary heart disease, myocardial infarction, angina, heart failure, or stroke, representing established cardiovascular conditions with prognostic significance.

## 3. Statistical analysis strategy

All statistical analyses were conducted using R statistical software, employing appropriate survey weights to account for the complex sampling designs of both databases. For NHANES analyses, mobile examination center (MEC) sampling weights were applied to generate nationally representative estimates for the United States population. Descriptive statistics were calculated with participants stratified by CMI quartiles, presenting continuous variables as means with standard deviations and categorical variables as frequencies with percentages.

Baseline characteristic comparisons across CMI quartiles employed appropriate statistical tests accounting for the complex survey design. For continuous variables, Kruskal-Wallis tests were utilized due to non-normal distributions commonly observed in epidemiological data. For categorical variables, Pearson chi-square tests with Rao-Scott adjustment were employed to account for survey design effects and clustering.

Survival analysis utilized Cox proportional hazards regression models to estimate hazard ratios (HR) and 95% confidence intervals (CI) for the association between CMI and mortality outcomes. Three sequential models were constructed to assess the robustness of associations: Model 1 included no covariates to assess crude associations; Model 2 adjusted for demographic and lifestyle factors including age, gender, education, BMI, smoking, and alcohol consumption; Model 3 represented the fully adjusted model incorporating all covariates including clinical comorbidities (hypertension, diabetes, cardiovascular disease).

CMI was examined both as a continuous variable and categorized into quartiles with the lowest quartile serving as the reference group. The proportional hazards assumption was assessed using Schoenfeld residuals and log-log survival plots, with no violations detected for the primary exposure variables.

Dose-response relationships were explored using restricted cubic spline (RCS) regression with three knots positioned at the 10th, 50th, and 90th percentiles of the CMI distribution. RCS models provide flexible assessment of non-linear associations while maintaining statistical power and interpretability. Tests for non-linearity were conducted using likelihood ratio tests comparing spline models to linear models.

Subgroup analyses were conducted to identify potential effect modifiers and vulnerable populations. Subgroups were defined by clinically relevant characteristics including gender, BMI status (<30 vs ≥ 30 kg/m^2^), smoking status, alcohol consumption, and presence of major comorbidities. Interaction terms were included in regression models to formally test for effect modification, with statistical significance set at *P* < .05 for interaction tests.

## 4. Ethical statement

This study utilized data from 2 publicly available databases: the NHANES and the CHARLS. The NHANES protocol was approved by the National Center for Health Statistics Research Ethics Review Board, and all participants provided written informed consent. The CHARLS protocol was approved by the Biomedical Ethics Review Committee of Peking University, and all participants provided written informed consent. As the present study involved secondary analysis of existing, fully de-identified public data and did not involve any direct interaction with human subjects, it was exempt from requiring additional ethical approval from our institutional review board, and the requirement for informed consent was waived for this specific analysis.

## 5. Results

### 5.1. Baseline characteristics and population demographics

The analytical populations comprised 1269 participants with depression from NHANES and 2997 participants with depression from CHARLS, representing substantial sample sizes for robust statistical inference. Baseline characteristics stratified by CMI quartiles are presented in Table 1, revealing important demographic and clinical patterns across cardiometabolic risk strata. In the NHANES cohort, participants demonstrated considerable heterogeneity across CMI quartiles. Mean age increased progressively from the lowest to highest CMI quartile (44.78 ± 16.39 years in Q1 to 50.41 ± 13.51 years in Q4, *P* = .009), indicating that higher cardiometabolic burden was associated with older age. Gender distribution showed significant variation across quartiles (*P* = .005), with the highest CMI quartile exhibiting a notable shift toward male predominance (48.02% male in Q4 vs 33.43% in Q1). Educational attainment remained relatively stable across quartiles (*P* = .5), suggesting that cardiometabolic risk stratification was independent of educational achievement in this depression population.

**Table 1:** Baseline characteristics of combined cohorts

	NHANES						CHARLS					
Characteristic	Overall, N = 1269 (100%)*	Q1, N = 307 (25%)*	Q2, N = 301 (25%)*	Q3, N = 335 (25%)*	Q4, N = 326 (25%)*	*P* value^2^	Overall, N = 2997 (100%)‡	Q1, N = 750 (25%)‡	Q2, N = 749 (25%)‡	Q3, N = 749 (25%)‡	Q4, N = 749 (25%)‡	*P* value†
**Age, (years**)	47.78 (15.46)	44.78 (16.39)	47.16 (16.58)	48.79 (14.66)	50.41 (13.51)	**.009**	59.94 (9.24)	60.60 (9.72)	59.89 (9.38)	59.80 (8.94)	59.47 (8.86)	.2
**Gender, *n* (%**)						**.005**						**<.001**
Female	817.00 (63.17%)	214.00 (66.57%)	196.00 (67.14%)	225.00 (67.00%)	182.00 (51.98%)		1859.00 (62.03%)	402.00 (53.60%)	451.00 (60.21%)	506.00 (67.56%)	500.00 (66.76%)	
Male	452.00 (36.83%)	93.00 (33.43%)	105.00 (32.86%)	110.00 (33.00%)	144.00 (48.02%)		1138.00 (37.97%)	348.00 (46.40%)	298.00 (39.79%)	243.00 (32.44%)	249.00 (33.24%)	
**Education, *n* (%**)						.5						.8
Below high school	461.00 (26.14%)	96.00 (24.08%)	100.00 (23.87%)	133.00 (29.39%)	132.00 (27.23%)		2864.00 (95.56%)	718.00 (95.73%)	720.00 (96.13%)	714.00 (95.33%)	712.00 (95.06%)	
High school or above	808.00 (73.86%)	211.00 (75.92%)	201.00 (76.13%)	202.00 (70.61%)	194.00 (72.77%)		133.00 (4.44%)	32.00 (4.27%)	29.00 (3.87%)	35.00 (4.67%)	37.00 (4.94%)	
**BMI, *n* (%**)						**<.001**						**<.001**
<30	639.00 (52.22%)	250.00 (84.23%)	158.00 (51.01%)	143.00 (42.49%)	88.00 (31.11%)		2871.00 (95.80%)	741.00 (98.80%)	729.00 (97.33%)	710.00 (94.79%)	691.00 (92.26%)	
>=30	630.00 (47.78%)	57.00 (15.77%)	143.00 (48.99%)	192.00 (57.51%)	238.00 (68.89%)		126.00 (4.20%)	9.00 (1.20%)	20.00 (2.67%)	39.00 (5.21%)	58.00 (7.74%)	
**Smoking status, *n* (%**)						.6						**<.001**
No	510.00 (36.07%)	141.00 (39.93%)	113.00 (33.73%)	133.00 (36.02%)	123.00 (34.60%)		1942.00 (64.80%)	442.00 (58.93%)	470.00 (62.75%)	523.00 (69.83%)	507.00 (67.69%)	
Yes	759.00 (63.93%)	166.00 (60.07%)	188.00 (66.27%)	202.00 (63.98%)	203.00 (65.40%)		1055.00 (35.20%)	308.00 (41.07%)	279.00 (37.25%)	226.00 (30.17%)	242.00 (32.31%)	
**Drinking status, *n* (%**)						.7						**<.001**
No	342.00 (21.02%)	81.00 (18.89%)	75.00 (19.84%)	99.00 (22.72%)	87.00 (22.65%)		2191.00 (73.11%)	457.00 (60.93%)	565.00 (75.43%)	591.00 (78.91%)	578.00 (77.17%)	
Yes	927.00 (78.98%)	226.00 (81.11%)	226.00 (80.16%)	236.00 (77.28%)	239.00 (77.35%)		806.00 (26.89%)	293.00 (39.07%)	184.00 (24.57%)	158.00 (21.09%)	171.00 (22.83%)	
**Hypertension, *n* (%**)						**<.001**						**<.001**
No	605.00 (51.43%)	186.00 (68.73%)	158.00 (58.29%)	152.00 (45.16%)	109.00 (33.51%)		1735.00 (57.89%)	499.00 (66.53%)	473.00 (63.15%)	420.00 (56.07%)	343.00 (45.79%)	
Yes	664.00 (48.57%)	121.00 (31.27%)	143.00 (41.71%)	183.00 (54.84%)	217.00 (66.49%)		1262.00 (42.11%)	251.00 (33.47%)	276.00 (36.85%)	329.00 (43.93%)	406.00 (54.21%)	
**Diabetes, *n* (%**)						**<.001**						**<.001**
No	944.00 (79.91%)	278.00 (94.17%)	239.00 (87.06%)	247.00 (79.03%)	180.00 (59.33%)		2724.00 (90.89%)	712.00 (94.93%)	700.00 (93.46%)	684.00 (91.32%)	628.00 (83.85%)	
Yes	325.00 (20.09%)	29.00 (5.83%)	62.00 (12.94%)	88.00 (20.97%)	146.00 (40.67%)		273.00 (9.11%)	38.00 (5.07%)	49.00 (6.54%)	65.00 (8.68%)	121.00 (16.15%)	
**CVD, *n* (%**)						.084						**<.001**
No	1014.00 (83.50%)	263.00 (86.57%)	248.00 (86.04%)	268.00 (83.70%)	235.00 (77.71%)		2462.00 (82.15%)	639.00 (85.20%)	640.00 (85.45%)	612.00 (81.71%)	571.00 (76.23%)	
Yes	255.00 (16.50%)	44.00 (13.43%)	53.00 (13.96%)	67.00 (16.30%)	91.00 (22.29%)		535.00 (17.85%)	111.00 (14.80%)	109.00 (14.55%)	137.00 (18.29%)	178.00 (23.77%)	

* Mean (SD); *n* (unweighted) (%).

† Kruskal Wallis test; Pearson χ^2^ test.

‡ Mean (SD); *n* (%).

The most striking differences were observed in metabolic parameters and clinical comorbidities. Obesity prevalence increased dramatically across CMI quartiles, from 15.77% in Q1 to 68.89% in Q4 (*P* < .001), highlighting the strong correlation between CMI and adiposity measures. Hypertension prevalence demonstrated a similar pattern, rising from 31.27% in Q1 to 66.49% in Q4 (*P* < .001). Diabetes prevalence showed the most pronounced gradient, increasing from 5.83% in Q1 to 40.67% in Q4 (*P* < .001), emphasizing the strong association between CMI and glucose metabolism disorders.

The CHARLS cohort exhibited comparable but distinct patterns reflecting the older age structure and different demographic characteristics of the Chinese population. Mean age remained relatively stable across CMI quartiles (ranging from 59.47 to 60.60 years, *P* = .2), likely reflecting the age-restricted sampling frame (≥45 years) and potentially different age-related cardiometabolic patterns in the Chinese population. Gender distribution showed significant variation (*P* < .001), with higher CMI quartiles associated with increased female representation, contrasting with the NHANES pattern and potentially reflecting cultural, genetic, or lifestyle differences between populations.

Educational patterns in CHARLS differed markedly from NHANES, with over 95% of participants having below high school education across all quartiles (*P* = .8), reflecting historical educational patterns in China and the older age structure of the cohort. Despite this educational homogeneity, clear gradients in metabolic comorbidities were observed. Diabetes prevalence increased from 5.07% in Q1 to 16.15% in Q4 (*P* < .001), while cardiovascular disease prevalence rose from 14.80% in Q1 to 23.77% in Q4 (*P* < .001). Notably, obesity rates remained relatively low across all quartiles compared to NHANES, with the highest prevalence of 7.74% in Q4, reflecting different obesity patterns in the Chinese population.

Behavioral risk factors showed interesting cross-cultural patterns. In NHANES, smoking and drinking behaviors showed no significant variation across CMI quartiles (*P* = .6 and *P* = .7, respectively), suggesting that these behaviors were not strongly associated with cardiometabolic risk in the depression population. Conversely, in CHARLS, both smoking and drinking showed significant associations with CMI quartiles (*P* < .001 for both), with higher CMI associated with lower rates of both behaviors, potentially reflecting cultural differences in substance use patterns or survival bias in the older Chinese population.

### 5.2. CMI and all-cause mortality associations

The primary analysis examining associations between CMI and all-cause mortality revealed complex patterns that differed significantly between the 2 study populations. In the NHANES cohort, Cox proportional hazards regression analyses demonstrated no statistically significant associations between CMI and all-cause mortality across all 3 analytical models. When CMI was examined as a continuous variable, the hazard ratios remained close to unity across all models: HR = 1.00 (95% CI: 0.95-1.06, *P* = .882) in the unadjusted Model 1, HR = 1.00 (95% CI: 0.93–1.07, *P* = .983) in the demographic-adjusted Model 2, and HR = 0.99 (95% CI: 0.91–1.07, *P* = .736) in the fully adjusted Model 3.

Quartile-based analysis in NHANES provided additional insights into the shape of the CMI-mortality relationship. Using the lowest quartile (Q1) as the reference group, none of the higher quartiles demonstrated statistically significant associations with all-cause mortality. In the fully adjusted Model 3, Q2 showed HR = 1.09 (95% CI: 0.61–1.94, *P* = .773), Q3 demonstrated HR = 0.74 (95% CI: 0.36–1.50, *P* = .401), and Q4 exhibited HR = 0.71 (95% CI: 0.34–1.46, *P* = .347). These results suggest a potential U-shaped or non-linear relationship, although the confidence intervals were wide and none reached statistical significance.

The CHARLS cohort presented similarly null findings for CMI-mortality associations, despite the larger sample size and longer follow-up period. Continuous CMI analysis revealed HR = 0.95 (95% CI: 0.89–1.01, *P* = .076) in Model 1, HR = 0.99 (95% CI: 0.94–1.04, *P* = .562) in Model 2, and HR = 0.97 (95% CI: 0.92–1.03, *P* = .292) in Model 3. Notably, the unadjusted model showed a marginally significant trend toward reduced mortality risk with higher CMI (*P* = .076), but this association was completely attenuated after adjustment for demographic and clinical covariates.

Quartile analysis in CHARLS confirmed the absence of significant associations, with all quartiles showing hazard ratios close to unity in the fully adjusted model: Q2 HR = 1.03 (95% CI: 0.77–1.37, *P* = .856), Q3 HR = 1.09 (95% CI: 0.81–1.47, *P* = .570), and Q4 HR = 0.95 (95% CI: 0.69–1.29, *P* = .721). The consistency of null findings across both populations and analytical approaches suggests that CMI may not serve as a robust predictor of all-cause mortality in depression populations, contrary to its established utility in general populations.

### 5.3. Dose-response relationships and non-linear associations

To comprehensively evaluate potential non-linear relationships between CMI and mortality outcomes, restricted cubic spline analyses were conducted for both cohorts. The RCS models provide flexible assessment of dose-response relationships without imposing linear assumptions, allowing detection of threshold effects, U-shaped relationships, or other complex patterns that might be obscured by linear modeling approaches.

**Figure 2.**
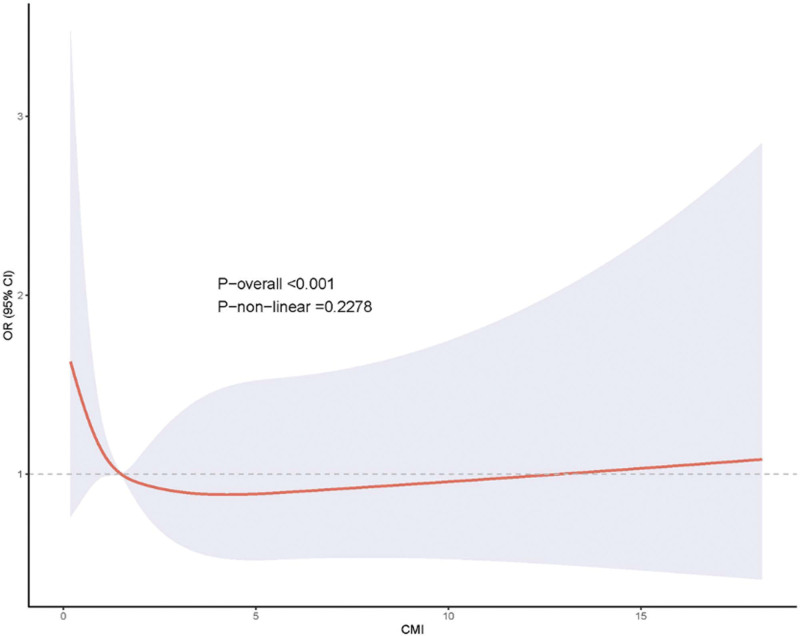
Restricted cubic spline analysis of all-cause mortality in NHANES.

The NHANES all-cause mortality RCS analysis, presented in Figure 2, demonstrated no significant overall association (*P*-overall < .001) but more importantly revealed no evidence of non-linear relationship patterns (*P*-non-linear = .2278). The spline curve remained relatively flat across the CMI distribution, with confidence intervals encompassing the null value throughout most of the range. This finding reinforces the conclusion that CMI does not demonstrate meaningful associations with all-cause mortality in the NHANES depression population, regardless of the analytical approach employed.

**Figure 3.**
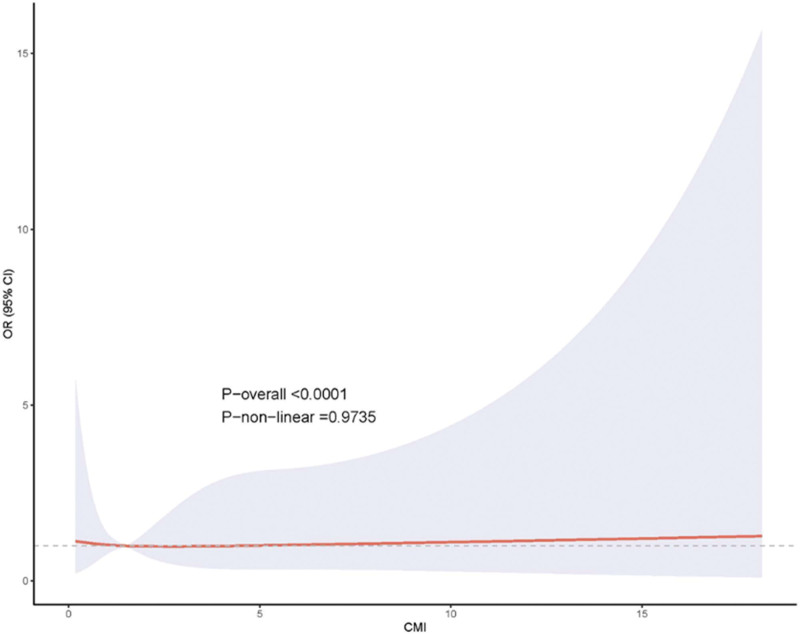
Restricted cubic spline analysis of cardiovascular mortality in NHANES.

Cardiovascular mortality analysis in NHANES, illustrated in Figure 3, similarly showed no significant non-linear patterns (*P*-non-linear = .9735), with the spline curve remaining close to the null value across the CMI distribution. The flat relationship pattern suggests that CMI does not capture cardiovascular-specific mortality risk in this population, despite its theoretical foundation based on cardiovascular risk factors.

**Figure 4.**
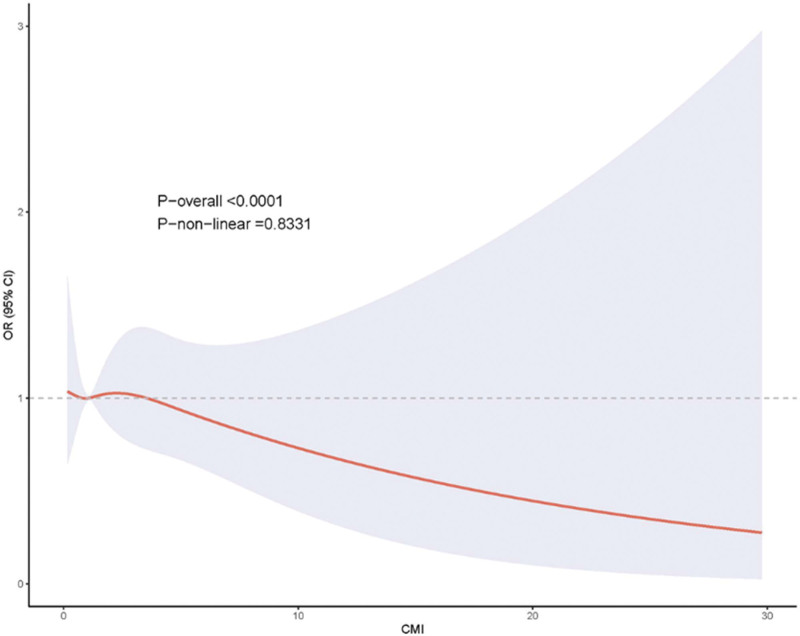
Restricted cubic spline analysis of all-cause mortality in CHARLS.

The CHARLS all-cause mortality RCS analysis, depicted in Figure 4, revealed an interesting pattern with significant overall association (*P*-overall < .0001) but no evidence of non-linearity (*P*-non-linear = .8331). The spline curve showed a generally declining trend with higher CMI values, consistent with the marginally protective effect observed in the unadjusted linear models. However, the absence of non-linear patterns suggests that this relationship, if real, follows a linear rather than threshold-based pattern.

The consistency of linear relationships across both populations and outcomes suggests that any true associations between CMI and mortality in depression populations would be detectable through standard linear regression approaches, supporting the validity of the primary Cox regression analyses and their null findings.

### 5.4. Subgroup analyses and effect modification

Comprehensive subgroup analyses were conducted to identify potential population segments where CMI might demonstrate stronger or different associations with mortality outcomes. These analyses are particularly important given the heterogeneity of depression populations and the potential for effect modification by demographic, behavioral, or clinical characteristics.

**Figure 5.**
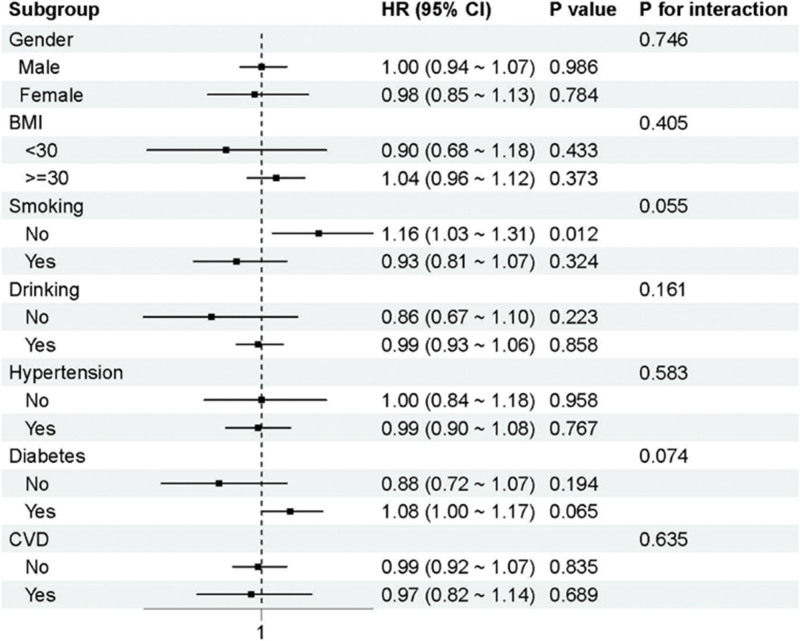
Subgroup analysis of all-cause mortality in NHANES.

The NHANES all-cause mortality subgroup analysis, presented in Figure 5, revealed several noteworthy patterns despite the overall null findings in the primary analysis. Among nonsmoking participants, CMI demonstrated a significant positive association with all-cause mortality (HR = 1.16, 95% CI: 1.03–1.31, *P* = .012), suggesting that cardiometabolic burden may be more strongly predictive of adverse outcomes in the absence of competing risk factors such as smoking. Although the formal interaction test did not reach statistical significance (*P*-interaction = .055), the marginally significant *P*-value suggests potential effect modification that warrants further investigation.

Gender-stratified analyses in NHANES showed no significant differences between male and female participants (*P*-interaction = .997), with comparable point estimates and confidence intervals across gender groups. BMI-stratified analyses similarly showed no evidence of effect modification (*P*-interaction = .712), suggesting that the relationship between CMI and mortality is not substantially modified by overall adiposity status. Diabetes and cardiovascular disease status also showed no significant interaction effects (*P*-interaction = .489 and .529, respectively), indicating that the presence of established cardiometabolic diseases does not substantially alter the CMI-mortality relationship.

**Figure 6.**
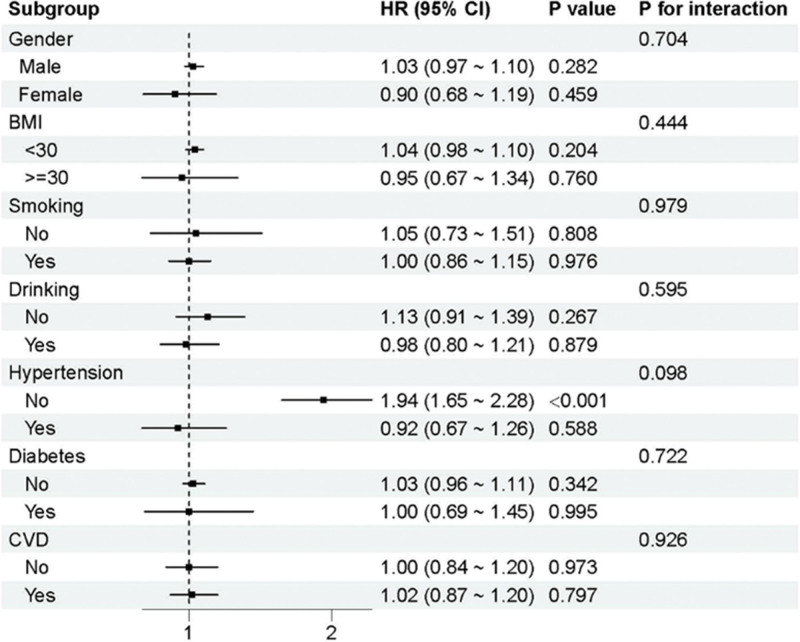
Subgroup analysis of cardiovascular mortality in NHANES

The NHANES cardiovascular mortality subgroup analysis, illustrated in Figure 6, revealed a particularly striking finding among participants without hypertension. In this subgroup, CMI demonstrated a strong positive association with cardiovascular mortality (HR = 1.94, 95% CI: 1.65–2.28, *P* < .001), representing one of the most robust associations identified in the entire analysis. The interaction test approached statistical significance (*P*-interaction = .098), suggesting that hypertensive status may indeed modify the relationship between CMI and cardiovascular outcomes. This finding has important clinical implications, suggesting that CMI may be most useful for cardiovascular risk stratification in depression patients without established hypertension.

**Figure 7.**
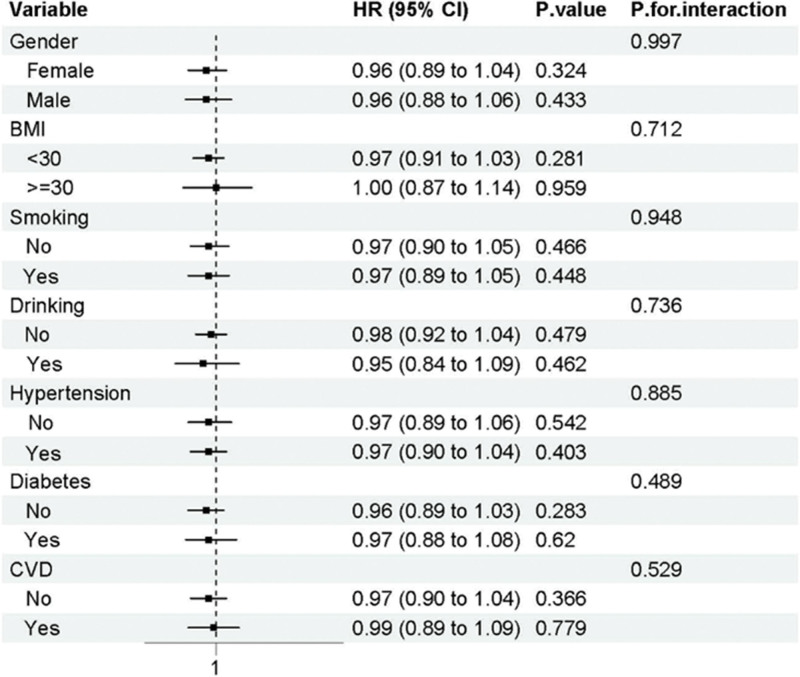
Subgroup analysis of all-cause mortality in CHARLS

The CHARLS all-cause mortality subgroup analysis, presented in Figure 7, showed remarkably consistent null findings across all examined subgroups. No subgroup demonstrated statistically significant associations between CMI and mortality, and all interaction tests yielded non-significant results (all *P*-interaction > .05). This consistency across subgroups strengthens the conclusion that CMI does not demonstrate meaningful prognostic utility for mortality prediction in the Chinese depression population studied in CHARLS.

The differential subgroup findings between NHANES and CHARLS highlight important population-specific differences that may reflect genetic, cultural, environmental, or healthcare system factors. The identification of specific high-risk subgroups in NHANES (non-smokers for all-cause mortality, non-hypertensive individuals for cardiovascular mortality) suggests that CMI may have selective utility in certain clinical contexts, supporting a more nuanced interpretation of the overall null findings.

## 6. Discussion

### 6.1. Principal findings and clinical implications

This comprehensive dual-database analysis represents the largest investigation to date examining the relationship between cardiometabolic index and mortality outcomes specifically in patients with depression. The study’s principal finding is the absence of consistent, statistically significant associations between CMI and mortality outcomes across 2 large, nationally representative cohorts from different continents and cultural contexts.^[1,2]^ But the identification of particular subgroups with strong associations implies a more nuanced relationship that has significant clinical implications for risk assessment stratagems targeting them.

The null findings in the main analyses differ from other work with clear evidence for associations between CMI and adverse outcomes in general populations.^[3,4]^ This difference might reflect the unique pathophysiology of depression in that classic cardiometabolic risk factors may function by different mechanisms or are dwarfed by depression-specific causes of mortality like suicide, drug toxicity, or complex behavior.^[5,6]^ Depression is associated with chronic low-grade inflammation, dysregulation of stress hormones, and autonomic dysfunction, which can interfere with the normal correlation between metabolic factors and cardiovascular outcomes.^[7,8]^

The subgroup analyses provide the most clinically informative results from this investigation. The discovery of substantial CMI-mortality relationships in non-smoking depression cases in NHANES confirms that cardiometabolic risk evaluation is most apt to be of maximally useful application in the absence of overwhelming competing risk factors.^[9]^ Smoking is a strong predictor of early death, and its exclusion may be able to expose other weaker metabolic risk factors to become significant predictors.^[10]^ This evidence provides further support for the risk stratification model in which the measurement of CMI can be given priority to the lower-risk depression group.

Similarly, the strong association of CMI with cardiovascular mortality in non-hypertensive NHANES subjects has important clinical implications.^[11,12]^ Hypertension is a recognized, easily measurable cardiovascular risk factor that may overwhelm risk prediction models. Absent from it, composite variables such as CMI may be incrementally informative in conveying information to guide preventive action. This finding suggests CMI measurement might be of most value as a screening instrument among depression patients with no established cardiovascular risk factors.

### 6.2. Cross-cultural and population-specific considerations

The differential findings between the NHANES and CHARLS populations indicate important limitations in the generalizability of cardiometabolic risk assessment tools to diverse populations.^[13,14]^ The NHANES cohort, a younger, more ethnically diverse US population, showed selective associations in some subgroups, while the CHARLS cohort, an older, homogeneous Chinese population, showed consistent null findings for all analyses.

Such differences may reflect several important determinants.^[15,16]^ First, ethnic variability in lipid metabolism, insulin sensitivity, and risk for cardiovascular disease can condition the predictive capacity of composite measures like CMI.^[17]^ Asian populations like the Chinese have non-constant body composition patterns with greater cardiovascular risk at lower BMI and non-consistent relationships between waist circumference and metabolic disturbances in comparison to Western populations.^[18]^

Second, cultural and lifestyle characteristics can modify the relationship between cardiometabolic indicators and outcomes of health.^[19]^ Common Chinese dietary behavior, different exercise activities, and distinct healthcare usage patterns may influence how the metabolic disturbance is expressed in terms of risk of death. Lower overall obesity prevalence in CHARLS compared with NHANES may also limit the dynamic range of CMI and reduce its discriminative ability.^[20]^

Third, cohort differences in age structure may affect outcomes.^[21]^ The sample from CHARLS was consistently older and middle-aged compared to NHANES, which had young adults. Processes of metabolic change with age, cross-classified mortality risks, and survival bias among older groups could temper the relationship between measures of cardiometabolic risk and outcomes. Use of different measures for depression, with NHANES employing the PHQ-9^[22]^ and CHARLS employing the CES-D-10,^[23,24]^ could also partially account for disparity in outcomes between the 2 groups.

### 6.3. Mechanistic considerations and pathophysiological insights

The theoretical foundation of CMI lies in its integration of central obesity (through waist-to-height ratio) and atherogenic dyslipidemia (through triglyceride-to-HDL ratio), both key components of metabolic syndrome and established cardiovascular risk factors.^[25]^ However, in this specific study, we observed a notable lack of consistent association between CMI and mortality risk in patients with depression. This finding prompts a critical inquiry into why traditional metabolic markers might lose their predictive power within this particular population.

A compelling explanation for the neutral or even paradoxical associations we observed, particularly the tendency toward lower mortality risk at higher CMI levels in some analyses, is the well-known “obesity paradox.” This phenomenon, widely documented in chronic conditions such as heart failure, suggests that higher adiposity or related indices do not uniformly translate into worse outcomes and can paradoxically be associated with survival benefits.^[26]^ In the context of depression, which is often characterized by chronic catabolic states or metabolic fluctuations, these indices may fail to capture the complex balance between nutritional reserves and systemic metabolic dysfunction.

Beyond the obesity paradox, depression is associated with multiple other pathophysiological changes that could significantly modify traditional cardiometabolic risk relationships.^[27]^ Chronic activation of the hypothalamic-pituitary-adrenal axis, for instance, leads to sustained cortisol elevation, promoting central adiposity and insulin resistance. However, this depression-related metabolic dysfunction may operate through different pathways than primary metabolic disorders, potentially altering the predictive utility of composite measures. Moreover, the unique pathophysiological landscape of depression also encompasses chronic low-grade inflammation and altered autonomic nervous system activity.^[5,28]^ These systemic changes can independently drive mortality or interact with traditional metabolic risk factors in ways that are not fully captured by composite indices like CMI, which were primarily designed for general populations.

The bidirectional relationship between depression and metabolic syndrome has been well-established in epidemiological studies, with each condition increasing the risk of developing the other.^[15]^ Nevertheless, the overall predictive signal of cardiometabolic markers in depressed individuals for all-cause mortality is further complicated by the potential for depression-specific causes of mortality, such as suicide or adverse effects of psychotropic medications.^[6,29]^ These factors can significantly dilute or mask the predictive power of cardiometabolic indices on overall mortality. Consequently, in a population facing such multifaceted health challenges and diverse mortality pathways, the prognostic utility of a broad metabolic index like CMI may be diminished, masked, or even altered by the predominant effects of the underlying psychiatric illness itself.

### 6.4. Methodological strengths and limitations

This investigation possesses several important methodological strengths that enhance the reliability and generalizability of findings. The utilization of 2 large, nationally representative databases provides substantial statistical power and enables cross-cultural validation of findings. The NHANES database represents one of the most rigorous population health surveillance systems globally, with standardized protocols, quality-controlled laboratory measurements, and comprehensive covariate assessment.^[13]^ Similarly, CHARLS represents the most comprehensive aging study in China, with protocols harmonized with international standards.^[2]^

The analytical approach employed multiple complementary strategies to ensure robust evaluation of CMI-mortality relationships. The use of both continuous and categorical CMI variables, sequential adjustment models, and restricted cubic spline analyses provides comprehensive assessment of potential linear and non-linear associations. The extensive subgroup analyses enable identification of clinically relevant population segments and potential effect modifiers. The validation of depression assessment instruments used in both databases, including the PHQ-9^[22]^ and CES-D-10,^[23]^ ensures reliable identification of depression cases across populations.

However, several limitations must be acknowledged. The observational study design precludes causal inference, and residual confounding by unmeasured factors remains possible. Depression assessment relied on validated questionnaire instruments rather than clinical diagnostic interviews, potentially leading to misclassification of depression status. The cross-sectional assessment of CMI at baseline does not account for changes in cardiometabolic status over time, which may influence long-term outcomes. The relatively short follow-up period in NHANES may be insufficient to capture long-term cardiometabolic effects, particularly in younger participants.

Mortality ascertainment methods differed between databases, with NHANES using National Death Index linkage and CHARLS employing interview-based methods, potentially introducing differential outcome misclassification. The definition of cardiovascular mortality was limited to specific ICD codes and may not capture all cardiovascular-related deaths, particularly sudden cardiac death or deaths where cardiovascular disease was a contributing but not primary cause. Additionally, the analysis did not account for competing risks from non-cardiovascular causes, which may be particularly relevant in depression populations with elevated suicide risk and other mental health-related mortality factors.

### 6.5. Clinical practice implications and future directions

Despite the null overall results, there are significant implications of this study for clinical practice as well as future research directions. That some subgroups were identified as having CMI-mortality relationships suggests that cardiometabolic risk screening in depressed patients must occur in a more subtle, individualized fashion, rather than in blanket application of risk prediction tools.^[12]^

For clinicians managing patients with depression, our findings reinforce that, in the absence of more robust depression-specific cardiometabolic risk tools, reliance on established, traditional cardiovascular risk assessment tools remains the cornerstone for overall risk stratification.^[11,12]^ However, while CMI did not demonstrate broad predictive utility in the overall depressed cohort, our subgroup analyses suggest that its evaluation may still provide incremental prognostic value in specific, lower-risk populations, particularly in non-smoking patients or those without established hypertension, where competing major risk factors do not obscure the metabolic signal.^[9,11]^ This incremental value would stem from its ability to capture central adiposity and dyslipidemia, which are still relevant metabolic perturbations, albeit less dominant in overall mortality prediction in this complex cohort.

Furthermore, our findings prompt a broader question about the optimal metabolic index for risk stratification in this population. For instance, the triglyceride-glucose (TyG) index, a simple and cost-effective surrogate marker of insulin resistance, has emerged as a robust predictor of adverse outcomes in various high-risk cardiovascular populations. Studies have shown its strong predictive value for long-term major adverse cardiovascular events and mortality in patients with high cardiovascular risk or established heart failure.^[30,31]^ Unlike CMI, which incorporates a measure of adiposity, the TyG index focuses purely on the interplay between glucose and lipid metabolism, which is central to insulin resistance. Future research should prioritize head-to-head comparisons of CMI, the TyG index, and traditional risk scores within depressed cohorts. Such studies would be crucial to determine whether any of these indices offer incremental prognostic value and to develop more tailored, effective risk-stratification strategies for this vulnerable population.

The results also highlight population-specific validation of risk prediction instruments. The different results in United States and Chinese populations illustrate the importance of cultural and ethnic tailoring of cardiometabolic risk assessment strategies.^[14]^ Health systems that have heterogeneous populations should give precedence to population-specific validation of risk prediction instruments rather than assuming universal applicability. The relationship of psychiatric disorder with body composition varies across populations and may influence the utility of anthropometry-based risk markers.^[19]^

Future research must prioritize several areas. Longitudinal studies with repeated CMI measurements could more precisely characterize the reciprocity between dynamic cardiometabolic change and mortality outcomes. Investigation of depression-specific cardiometabolic risk factors, including inflammation markers, stress hormones, and medication effects, might create more appropriate risk prediction tools for this population.^[5,27]^ The interaction of depression with conditions such as chronic obstructive pulmonary disease, which also influences risk of mortality, requires further investigation.^[32]^

Intervention studies examining whether treatment plans informed by CMI improve outcomes in depressed patients would provide instructive evidence of clinical usefulness. Genetic studies of population-specific variations in cardiometabolic risk could also inform personalized risk assessment approaches. Development of depression-specific risk prediction models incorporating mental health-specific factors alongside traditional cardiometabolic variables is an important research priority. These can include depression severity, treatment response, medication effects, and psychosocial factors that are not captured by traditional cardiometabolic measures.^[6,29]^

## 7. Conclusion

This comprehensive analysis of cardiometabolic index and depression patient mortality outcomes in 2 big international databases provides important evidence on the utility of combined cardiometabolic risk assessment in this vulnerable population.^[1,2]^ While the primary findings demonstrate an absence of consistent associations between CMI and mortality outcomes, the finding of significant relations in specific subgroups points to a more complex picture requiring individualized risk assessment approaches.

The results indicate that standard cardiometabolic risk prediction tools will have limited applicability in populations with depression, possibly due to the unique pathophysiology of depression and its interaction with metabolic dysfunction.^[5,15]^ Selective application in specific clinical contexts, nonetheless, particularly in patients without overwhelming competing risk factors, can provide valuable clinical insight. The differential findings among Chinese and United States populations highlight the supreme significance of cross-cultural validation of risk prediction instruments and warrant the development of population-specific approaches to cardiometabolic risk screening.^[13,14]^

The findings contribute to the growing appreciation of depression as a complex systemic illness requiring specialist approaches to risk assessment and management.^[16,21]^ The analysis demonstrates that while CMI cannot be used as a universal mortality predictor in depression populations, its utility may be population-specific and context-dependent. This nuanced understanding is relevant to clinicians who must navigate the complex interplay between mental illness and cardiometabolic risk in their patients, particularly in view of established associations between depression and metabolic disease.^[8,26]^

The study also emphasizes the necessity of adjusting for competing risk factors in the evaluation of novel biomarkers in high-risk populations.^[9,10]^ The fact that there were significant associations in some subgroups, such as non-smokers and those without hypertension, suggests that the clinical utility of CMI can be optimized with selective application rather than universal screening approaches. This finding is in line with the overall principle of interaction of classical cardiovascular risk factors and depression-associated risks of mortality.^[11,12]^

Furthermore, the cross-cultural findings highlight the necessity of population-specific validation of risk prediction tools, particularly within the framework of a globalized healthcare system wherein evidence-based interventions must be translated to populations with varying genetic, cultural, and environmental backgrounds.^[19]^ The Chinese sample’s uniform null findings compared to selective positive findings in the United States sample demonstrate that biomarker operation may vary significantly across ethnic and cultural populations, with the requirement to use validated measures of assessment appropriate for the population.^[22–24]^

The mechanistic results of this investigation also shed light on why metabolic disturbance and inflammation in depression may differ from primary cardiometabolic disease.^[7,28]^ The interrelationships between depression, components of metabolic syndrome, and cardiovascular events require further examination to maximally inform risk stratification strategies.^[18,25]^ The role of medication effects in modifying these relationships is also worthy of additional attention, given that antidepressants and other psychotropic medications exert a significant influence on weight and metabolic parameters.^[6,29]^

Lastly, this research underscores the need for continued investigation into depression patient risk stratification methods that are optimally effective, with an emphasis on the development of depression-specific tools that account for the unique biological, psychological, and social determinants of outcomes in this patient population.^[20]^ Future research must place high importance on the development of composite risk models for the evaluation of depression-specific factors alongside traditional cardiometabolic factors, with careful population-specific validation and implementation approaches.^[3,4]^ The ultimate objective is to provide clinicians with robust, evidence-based tools that can effectively screen for patients at high risk of depression and guide focused intervention to improve both mental and cardiometabolic health, recognizing the complex interplay between psychiatric disease and physical well-being in diverse populations.^[17,32]^

## Author contributions

**Formal analysis:** Yixuan Xie, Jiawang Jin, Ting Zhou.

**Methodology:** Yixuan Xie.

**Software:** Yixuan Xie, Jiawang Jin, Ting Zhou.

**Supervision:** Yixuan Xie.

**Writing – original draft:** Yixuan Xie, Jiawang Jin, Ting Zhou.

**Writing – review & editing:** Yixuan Xie.
